# A Virtual Health Library for Dementia Patients and Caregivers to Improve Quality of Information and Communication

**DOI:** 10.1093/geroni/igaa057.802

**Published:** 2020-12-16

**Authors:** Kirsten Mengell, Alyssa Indelicato, Sarah Lawler, Nancy Smith, Sharon Brangman, Telisa Stewart

**Affiliations:** 1 SUNY Upstate Medical University, Brewerton, New York, United States; 2 SUNY Upstate Medical University, Syracuse, New York, United States; 3 Nancy Smith Consulting, Syracuse, United States; 4 SUNY Upstate Medical University, Syracuse, United States

## Abstract

Access to accurate health information is critical for patient decision making and health communication. Unfortunately, there is limited quality health information for dementia patients and their caregivers. A Virtual Health Library (VHL) provided access to credible health information through the electronic medical record (EMR) to reach the dementia patients and their caregivers. A VHL was created and brought together clinicians, caregivers, technology support personnel, public health professionals, and the health sciences library. The team identified areas of interest and met monthly to create VHL materials. Materials included voice-over slides and 1-page educational content that was uploaded to the EMR for patient and caregiver access. A baseline and final questionnaire assessed demographics, empowerment, and shared decision making for both the patient and the caregivers and a pre/post was created for each module to asses knowledge and stratification. Initially, 1331 patients with dementia were recruited for the project from a university geriatrics department. The population had a 28.3% enrollment in the EMR and only 3.8% used the EMR in the past six months. Of this pool, during the initial launch 32 patients and or caregivers completed the baseline within the first week. 98% of respondents were caregivers with an average age of 58.7. With the youngest caregiver 42 and the oldest 88. Of the caregiver’s relationships to the patients is 43.3% their child, 40% their spouse, 10% other family, and 6.7% friends. During the project, participants improved access to health information and became empowered to engage with their healthcare provider.

